# Non-Intrusive Load Monitoring Approaches for Disaggregated Energy Sensing: A Survey

**DOI:** 10.3390/s121216838

**Published:** 2012-12-06

**Authors:** Ahmed Zoha, Alexander Gluhak, Muhammad Ali Imran, Sutharshan Rajasegarar

**Affiliations:** 1 Center for Communication Systems Research, University of Surrey, Guildford, GU2 7XH, UK; E-Mails: a.gluhak@surrey.ac.uk (A.G.); m.imran@surrey.ac.uk (M.A.I.); 2 Department of Electrical and Electronic Engineering, University of Melbourne, Melbourne, VIC 3010, Australia; E-Mail: r.sutharshan@ee.unimelb.edu.au

**Keywords:** Non-Intrusive Load Monitoring (NILM), Intrusive Load Monitoring (ILM), disaggregation algorithms, load signatures, energy management

## Abstract

Appliance Load Monitoring (ALM) is essential for energy management solutions, allowing them to obtain appliance-specific energy consumption statistics that can further be used to devise load scheduling strategies for optimal energy utilization. Fine-grained energy monitoring can be achieved by deploying smart power outlets on every device of interest; however it incurs extra hardware cost and installation complexity. Non-Intrusive Load Monitoring (NILM) is an attractive method for energy disaggregation, as it can discern devices from the aggregated data acquired from a single point of measurement. This paper provides a comprehensive overview of NILM system and its associated methods and techniques used for disaggregated energy sensing. We review the state-of-the art load signatures and disaggregation algorithms used for appliance recognition and highlight challenges and future research directions.

## 1. Introduction

Today, energy conservation is a challenging issue due to exponentially increasing energy demands. Researchers are striving to develop technological solutions in order to address this problem. In the European Union, the residential sector alone accounts for 30% of electricity usage. This is a growing concern as energy resources are limited and it is predicted that global energy demands will double by the end of 2030 [1] with negative implications on the environment (e.g., CO_2_ emissions). Energy crisis, climate change and the overall economy of a country is directly affected by the growth in energy consumption. A significant reduction in the energy wastage can be achieved through fine-grained monitoring of energy consumption and relaying of this information back to the consumers [2,3]. A detailed review [3] of more than 60 feedback studies suggest that maximum energy saving can be achieved using direct feedback mechanisms (*i.e.*, real-time appliance level consumption information) as opposed to indirect feedback mechanisms (*i.e.*, monthly bills, weekly advice on energy usage). Motivated by this, we see a large scale deployment of smart meters in the residential environment by the governments of UK and USA. While it is envisioned that the smart meters will charge consumers based on peak or off peak timings [4], traditional smart meters are only able to measure energy consumption data at a house level granularity. In order to implement a precise demand-response functionality, a much finer granularity of information is required. To achieve this, research efforts have led to the development of Appliance Load Monitoring (ALM) methods.

The goal of ALM is to perform detailed energy sensing and to provide information on the breakdown of the energy spent. This would further enable the automated energy management systems to profile high energy consuming appliances, allowing them to devise energy conservation strategies such as re-scheduling of high power demanding operations for the off-peak times. Moreover, companies would be able to develop a better understanding of the relationship between appliances and their usage patterns. The concept of ALM is decades old but lately we have seen a growing interest in this research area inspired by parallel advancements in sensing technology, data communication and networks, artificial intelligence and machine learning methods. ALM is an essential prerequisite for providing energy feedback to the residential consumers, but it is equally beneficial for the industrial sector because of its applicability in fault detection and remote load monitoring services.

There are two major approaches to ALM, namely Intrusive Load Monitoring (ILM) and Non-Intrusive Load Monitoring (NILM). In the literature, ILM and NILM are alternatively referred to as distributed sensing and single point sensing methods respectively. This is because the ILM approaches require one or more than one sensor per appliance to perform ALM, whereas NILM just requires only a single meter per house or a building that is to be monitored. Although the ILM method is more accurate in measuring appliance-specific energy consumption compared with NILM, the practical disadvantages includes high costs, multiple sensor configuration as well as installation complexity favoring the use of NILM especially for the case of large scale deployments. Consequently, established as well as start-up companies along with academic researchers have focused their attention on the improvement of NILM based approaches [5] in order to make it a viable solution for a realistic environment. Motivated by this, we provide a comprehensive discussion on the appliance signatures and load identification algorithms used in NILM for disaggregated energy sensing.

The remainder of the paper is organized as follows. In the next section, we provide a brief introduction to the NILM framework, whereas we discuss in detail the state-of-the art appliance features used for energy disaggregation in Section 3. In Section 4 we present recent advances as well as insights into load disaggregation techniques being applied in NILM by providing a comparison of several learning algorithms as well as highlighting their limitations. In Section 5, we discuss performance evaluation metrics to assess the accuracy of NILM systems. Furthermore, we give an account of the current practices beyond traditional NILM methods to improve the overall appliance disaggregation accuracy, and summarize the prevailing challenges and future research directions in Section 6. Finally, we conclude in Section 7.

## 2. General Framework of NILM

**Figure 1.**
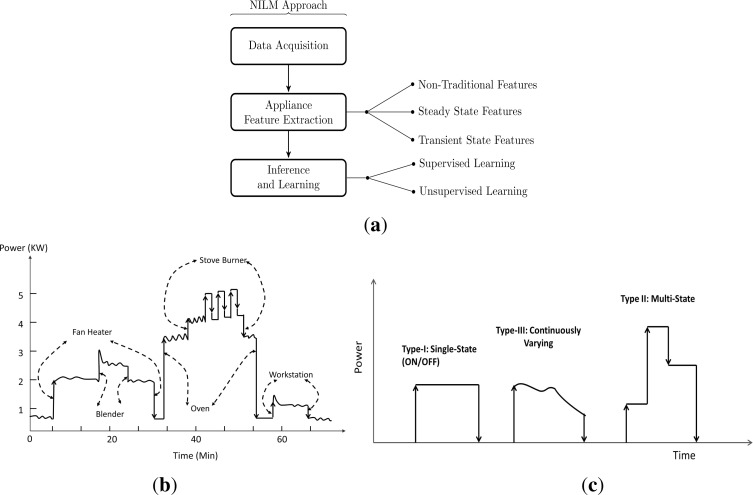
(**a**) General framework of NILM approach (**b**) An aggregated load data obtained using single point of measurement; (**c**) Different load types based on their energy consumption pattern.

In this section, we provide an introduction to a general framework for NILM system as shown in Figure 1(a). The concept of NILM is not new as almost two decades back Hart [6] proposed a method for disaggregating electrical loads by examining only the appliance specific power consumption signatures within the aggregated load data. The data is acquired from the main electrical panel outside the building or the residence, hence it is considered to be non-intrusive as the method avoids any equipment installation inside the customer’s property. The goal is to partition the whole-house building data into its major constituents. This problem can be formulated as follows: The power signals from the active appliances aggregate at the entry point of the meter as *P* (*t*) as shown in Figure 1(b), where this can be mathematically defined as

(1) $$P(t)=p_{1}(t)+p_{2}(t)+\ldots +p_{n}(t)$$

where *p_i_* is the power consumption of individual appliances contributing to the aggregated measurement and *n* is the total number of active appliances within the time period *t*. The task of the NILM is to perform decomposition of *P* (*t*) into appliance specific power signals in order to achieve disaggregated energy sensing. Electrical loads exhibits a unique energy consumption pattern often termed as “load or appliance signatures”, that enables the disaggregation algorithms to discern and recognize appliance operations from the aggregated load measurements. Appliance identification is highly dependent on load signatures, which are further characterized by the appliance category. As proposed by [6], consumer appliances can be categorized based on their operational states as follows:

1. Type-I: These are the appliances with only two states of operation (ON/OFF). Examples of such devices includes table lamp, toaster, *etc*.
2. Type-II: These are multi-state appliances with a finite number of operating states also referred to as Finite State Machines (FSM). Consumer appliances belonging to this category includes washing machine, stove burner *etc*. The switching pattern of these appliances is also repeatable, which makes it easier for the disaggregation algorithm to identify their operation.
3. Type-III: The appliances belonging to this category are also known as Continuously Variable Devices (CVD) because of their variable power draw characteristics with no fixed number of states. The power drill and dimmer lights are examples of CVD’s with no repeatability in their power draw characteristics. Hence it is very challenging for the NILM methods to disaggregate these type of appliance from the aggregated load measurements.
4. Type-IV: In [5,7] authors have highlighted another category of appliances that remain active throughout weeks or days consuming energy at a constant rate and therefore referred to as *“permenant consumer devices”*. Appliances such as hardwired smoke detector, telephone sets, cable TV receivers are amongst the devices belonging to this category.

The energy consumption pattern of different type of loads have been shown in Figure 1(c), which is further translated as an appliance feature to distinguish between different appliance categories. Research to date has tended to focus on defining load signatures tailored to the appliance categories listed above in order to characterize them in a best possible way for identification. However recently in [8] author has argued that appliances can have a multi-working model based on user customization and working styles, therefore this must be accounted into feature extraction process.

We will now briefly discuss the constituent modules starting from data acquisition to appliance recognition that define a general NILM framework as shown in Figure 1(a).

**Data Acquisition Module:** The role of the data acquisition module is to acquire aggregated load measurement at an adequate rate so that distinctive load patterns can be identified. There is a wide variety of power meters designed to measure the aggregated load of the building [9] that can be further classified as follows [8].

1. Low-Frequency Energy Meters: The commercial solutions available in the market today offer a range of sampling frequencies for the meters. The sampling rate determines the type of information that can be extracted from the electrical signals. In order to capture the higher order harmonics of the electrical signals, which are integral multiples of fundamental frequency (*i.e.*, 60 Hz), the sampling rate of the energy meter must fulfill the Nyquist–Shannon sampling criteria. For example, an energy meter such as Itron has a sampling rate of 600 Hz, this enables it to capture up to the 5th harmonic of the electrical signals (*i.e.*, 300 Hz). On the other hand, traditional power metrics such as real power, reactive power, Root Mean Square (RMS) voltage and current values can be computed at a low sampling rate (*i.e.*, 120 Hz). The computed metrics are either reported to the backend server via a Network Interface Card (NIC) or processed inside the meter. The high end NICs can read, write and report data up to 1 kHz, however changes are required in the meter hardware to support sampling rate greater than 5 kHz [10].
2. High-Frequency Energy Meters: In order to capture the transient events or the electrical noise generated by the electrical signals, the waveforms must be sampled at a much higher frequency in a range of 10 to 100 MHz. These types of high frequency energy meters are often custom-built and expensive due to sophisticated hardware and are tailored to the type of features that needs to be extracted from the signal.

Researchers however argue that most of the commercially available meters show a variation of 10% to 20% in data measurements [5]. In addition, low-cost metering solutions offer limited functionality as they are equipped with low resolution Analog to Digital (A/D) converter and small size on-chip Flash memory used by the processing unit for storing results after various operations [10]. Hence, in order to achieve medium or higher rate sampling of the electrical signals, researchers have started to developed their own prototypes for experimental evaluations as discussed in [5,9,11]. The data acquisition for NILM can further be categorized into whole-house and circuit level data. The typical NILM system makes use of whole-house data acquired from a single meter. However, one limitation of such an approach is that the identification of low-power and variable appliances in the presence of high-power loads from the whole-house data, which often becomes quite challenging. An alternative approach has been proposed by [12] to make use of the circuit-level power measurements, as it is often the case that high-power appliances receive a dedicated circuit within homes. The task of power decomposition becomes much easier as there are fewer devices on each circuit in contrast to the whole-house NILM, but at the expense of increased installation complexity and cost.

**Feature Extraction:** The next step after the data acquisition is to process the raw data (*i.e.*, voltage and current waveforms) in order to compute the power metrics (e.g., active and reactive power). The subsequent step after processing the raw data is to detect events such as appliance state transition (e.g., On to OFF) from the power measurements. An event detection module detects the ON/OFF transition of appliances by analysing the changes in power levels. These events can further be defined in terms of steady-state or transient changes, accordingly. In the literature, several event detection methods have been proposed [9,13–15] and in order to characterize the detected events, steady-state and transient event-based feature extraction methods are developed. Steady state methods identify devices based on variations in their steady state signatures, for example a change of steady-state active power measurement from a high to low value can identify whether the appliance is being turned On or Off. The transient methods on the other hand make use of transient signatures that uniquely define appliance state transitions by extracting features like shape, size, duration and harmonics of the transient waveforms. However, distinctive transient signatures can only be extracted if the sampling rate is higher than 1, 000 samples per second [5]. There has been a debate considering the use of either steady-state or transient based features extraction methods for load disaggregation as both of these approaches have their advantages and disadvantages. Bearing in mind the cost of the solution, the steady state methods seem to be a more feasible approach because it requires low-cost hardware. On the other hand, load disaggregation algorithms can incorporate transient features to improve the segregation of appliances with overlapping steady-state features, but at the cost of expensive hardware. Apart from event-based approaches, research efforts have also been made to completely avoid the event detection step either by making use of raw current and voltage readings [16] or by analysing the information in the frequency spectrum in order to detect the presence of certain appliances while they are being operated [17]. A detailed discussion on these methods is provided in Section 3.

**Load Identification:** The extracted appliance features are further analyzed by the load identification algorithms in order identify appliance-specific states from the aggregated measurement. Most of the research work in NILM method is focused on supervised machine learning techniques that require labeled data for training the classifier. To date, most of the supervised learning methods adapted for load disaggregation are either optimization based or pattern recognition based approaches. The optimization approach tries to match the observed power measurements *P* (*t*) to a possible combination of appliance power signals (present already in the database) to reduce the matching error as reported in [5,18,19]. However, one major drawback is that the presence of unknown loads in *P* (*t*) complicates the optimization problem as the method attempts to provide a solution based on the combination of known appliances [6,18]. Therefore the pattern recognition approach has been a preferred method by researchers for the task of load identification. Similar to pattern matching, extracted features are matched with a pool of load signatures already available in the appliance feature database in order to identify an event associated with a operation of an appliance. The requirement of training data for the algorithms is one of the major obstacles in the wide adoption of NILM solutions as discussed below. Recently, researchers have shown an increased interest in unsupervised methods for the load disaggregation so that the need for data annotation can be eliminated. Unlike most of the supervised load disaggregation approaches that rely on detection of events for classification, the unsupervised methods are non-event-based. These methods make use of unsupervised learning techniques and attempts to disaggregate the aggregated load measurements directly without performing any sort of event detection. We have provided a detailed discussion on the supervised and unsupervised learning algorithms used for the task of load disaggregation in Section 4.

**System Training:** The system training or a pre-learning phase is often a prerequisite for NILM systems. The supervised disaggregation algorithms need adequate labeled data for learning the model parameters in order to perform the task of appliance recognition. The training methods can further be classified into on-line or off-line. In case of on-line training, researchers [9,20] have used the time slice or window based methods for real-time detection and learning of appliance features. However, upon detection of load events manual labeling of the appliances is challenging and complex as only the aggregated load values are observed instead of individual appliance measurements. In order to facilitate the online training process, several sensor assisted training mechanisms are proposed in [21–23]. Conversely the off-line training approach acquire the aggregated load measurements from the target environment for a time period, such as for specific days or months as reported in [24], and appliances are labeled based on a pre-existing signature in the database. Alternatively, a sequential training method can also be employed in which the user manually changes the appliance states one by one to complete the appliance feature database [6], however this is very time consuming.

In order to ease the data annotation process, sub-metering approach has also been utilized [13,25,26] that requires installation of one energy meter per appliance to record appliance-specific consumption patterns. However, it not only incurs extra cost but also requires a complex installation of sensors on every device of interest, which is not feasible for large scale deployment of NILM systems. A possible solution to this problem has been addressed in [27], in which a simulation platform for an energy-aware smart metering system has been proposed with an aim to expedite the design process for complex smart metering solutions. Although the core focus of the simulator is to enable the designers to optimize the architecture and communication aspects of the system, it further provides an opportunity to simulate the behavior of various appliances within a house. The researchers working in the field of NILM can greatly benefit from this extra functionality as it could be used to analyze power dissipation patterns of various appliances and their interactions beforehand without the need for a real-world setup. The whole process of building an appliance signature database and data annotation is a tedious process and requires human intervention and supervision, whereas currently there are no standard automated solutions that can facilitate the training process. This has been one of the limiting factor that has hindered the widespread success of NILM solutions. Researchers [21,28] envision to build generic appliance signature databases, to be shared in a cloud for future applications in order to move ahead towards a unified framework. In [29], different training schemes for NILM have been compared and analyzed, and the associated challenges with each one have been highlighted.

**Figure 2.**
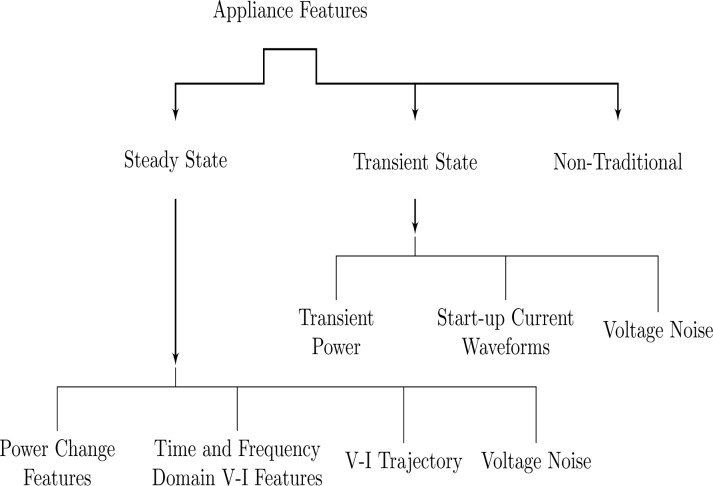
Taxonomy of appliance features for energy disaggregation.

Appliance features, as discussed earlier, can be broadly categorized into steady state and transient state features, however there are also non-traditional signatures that are often used in combination with traditional appliance features to improve the performance of load disaggregation algorithms. Steady-state analysis considers the stable states of appliance operation whereas transient analysis takes into account the transitional state during which the appliances power consumption behavior is unstable. Further subdivision of these two categories is shown in Figure 2. In this section, we will provide a detailed discussion on the appliance features used in the NILM systems.

## 3. Appliance Features for Energy Disaggregation

### 3.1. NILM Methods Based on Steady-State Analysis

The NILM methods based on steady-state analysis make use of steady-state features that are derived under the steady-state operation of the appliances. Real power (*P*) and Reactive power (*Q*) are two of the most commonly used steady state signatures in NILM [6] for tracking On/Off operation of appliances. The real power is the amount of energy consumed by an appliance during its operation. If the load is purely resistive then the current and voltage waveforms will always be in phase and there will be no reactive energy. For a purely reactive load the phase shift will be 90°, and there will be no transfer of real power. On the other hand, due to inductive and capacitive elements of the load, there is always a phase shift between current and voltage waveforms that generates or consumes a reactive power respectively.

**Figure 3.**
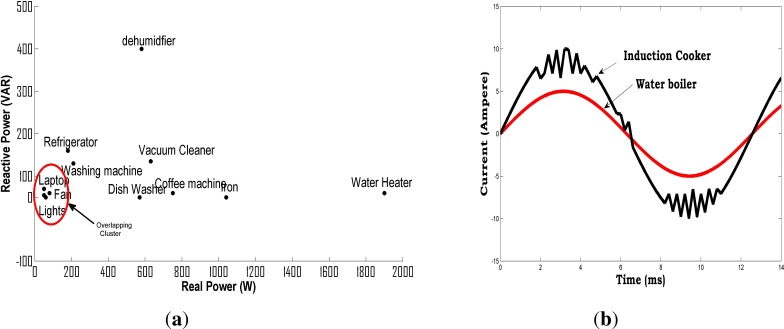
(**a**) Load distribution in *P*-*Q* Plane, (From [31]); (**b**) Current draw of linear *vs* non-linear loads, (From [18]).

Researchers [13,26,30] have tried to disaggregate load using real power as a single feature and found out that high-power appliances with distinctive power draw characteristics such as electrical heaters and water pumps can be easily identified from the aggregated measurements. However this method does not take into account appliances with similar power draw characteristics. In addition, simultaneous state transitions of appliances leads to erroneous results. In order to address some of these issues, it has been shown in [6,24] that the high power type-I and some of the type-II appliances can easily be differentiated by analyzing the step changes in real and reactive power features. At the same time, it is challenging for the power change method to discern appliances that exhibit overlapping in the *P-Q* feature space especially the low-power appliances as illustrated in the Figure 3(a).

In order to overcome the limitations of power based methods, researchers [32,37,42] have tried to analyze the current *I* and the voltage *V* waveforms and extracted unique appliance specific features such as peak and Root Mean Square (RMS) current and voltage values as well as the phase difference *φ* and Power Factor (*P F*) information to uniquely define an appliance activity. The power factor is simply a ratio between real and apparent power (which is the product of *I_RMS_* and *V_RMS_*) and it often varies from 1 to 0 depending on whether the load is motor-driven or resistive. These time-domain V-I features have shown good performance when employed within Real Time Recognition and Profiling of Appliances (RECAP) system [37] for the identification of On/Off operation of kitchen appliances. The RECAP system was the first systematic effort to integrate appliance profiling and recognition under a single framework, however it was acknowledged that these steady-state features are not suitable for recognizing multi-state appliance operations. The effectiveness of time domain features in recognizing various loads has also been demonstrated through experimental evaluations by [33]. It was found that the root mean square features are more discriminative in comparison with peak values, however, the experimental dataset does not include type-III appliances. Moreover, there is no discussion on the detection of simultaneous appliance activation sequences. In [18,34–36,38] authors have reported the use of Fourier series analysis to determine input current harmonics. In [38], it has been shown that most of the resistive loads have constant power (CP) whereas the switching loads have constant impedance (CI). Both types of loads can be characterized by their distinctive steady-state current harmonics; hence a method has been proposed to decompose the power signal by estimating the proportion of CP to CI using the frequency representation of current signals.

The current harmonics on the other hand can also uniquely characterize non-linear loads that draw non-sinusoidal current during the operation. It can easily be seen from Figure 3(b) that the water boiler has a sinusoidal current draw in contrast to the induction cooker, whereas higher order harmonics in the current waveform of induction cooker are quite easily visible. The harmonics have been used in combination with real and reactive power features [17,32] to improve the performance of the detection algorithm, however harmonic analysis requires high rate sampling of the waveforms. It was shown by [17] that appliances operating in parallel have unique steady-state harmonic signatures for each of their respective combination. Although this approach is suitable to recognize Type I and Type IV loads, to perform load identification it requires the availability of unique sets of harmonic signatures with respect to all possible device combinations. In [39,40] the author proposed a novel method of using V-I trajectory to categorize a group of appliances. For each appliance the V-I trajectory has been plotted using the normalized current and voltage values. The V-I trajectory separates the category of appliances into eight groups with high accuracy, providing further sub-division within each group. It has been shown that V-I based approach is more effective than existing approaches based on power measurements, for building a taxonomy of electrical appliances due to their distinct V-I curves.

Gupta *et al*. [41] proposed an interesting approach in which it has been shown that appliances equipped with Switch Mode Power Supply (SMPS) can be characterized by analyzing steady-state voltage noise generated upon their operation. However, the drawback is it not only requires additional hardware for measurement, but also this method is sensitive to the wiring architecture of the monitored environment. In Table 1, we provide a summary of our discussion on steady-state NILM methods and highlight the advantages and disadvantages of different approaches.

### 3.2. NILM Methods Based on Transient-State Analysis

The transient behavior of major appliances is found to be distinct and their features are less overlapping in comparison with steady state signatures, however the major limitation is the high sampling rate requirement in order to capture the transients [42]. Norford and Leeb [13] have shown that the shapes of transient events can also be used as a feature for appliance detection. Chang *et al.*[43] later demonstrated that the energy calculated during a “turn on” transient event could also be used to discriminate between appliances. In [44], the author used power spikes or overshoots during the transitional stage of the device as a feature to detect devices but the drawback is that they are appliance specific. Though these approaches were proven to be effective for load disaggregation, repeatability of transient events and high sampling rate requirement are the major drawbacks. In addition, appliances with similar transient characteristics are not differentiable by this approach.

The concept of analyzing the spectral envelope of the waveform based on Short-Time Fourier Transform (STFT) [14] was found to be quite useful in detecting variable loads along with other appliances. However, the main purpose of load monitoring is not only to detect appliances, but also to measure their energy consumption as well. Therefore in [45,46] researchers correlate spectral envelopes with *P* and *Q* components to address this problem. However, the robustness of the combined feature set has not been evaluated in the presence of unknown loads. Another limiting factor is that the proposed method demands excessive training of the system. In comparison with Fourier Transform, the wavelet transform has also been used to characterize the transient physical behavior of the loads. It has been shown by the author in [47] that the transient response time and the transient energy features are better than steady-state features for the task of appliance disaggregation; however the study considers selective appliances with distinct turn-on characteristics.

The recent work in NILM by Patel *et al.*[11,31] shows promising results using high-frequency (HF) sampling of voltage noise that occurs during the transient events (*i.e.*, switching from on to off).

The main concept is that each appliance emits voltage noise back to the main line. This is mainly true for appliances equipped with SMPS that create electro-magnetic interference. These noises are categorized into three types: on-off transient noise, steady-state line voltage noise, and steady-state continuous noise. These noises can be measured from any electrical outlet inside the home. The problem however with the current device recognition system is that, in order to measure the reactive and real power, it requires knowledge of the phase angle between the AC voltage and current, which can be measured using magnetic sensors. This requires installation of these sensors at a metering point by a professional electrician, therefore as a remedy to the problem Patel developed a prototype of a plug-in module that can be inserted into any wall socket. The study reveals that on-off transient noise signatures remain stable over time and can be used to identify unique sources of energy consumption (*i.e.*, light bulb in room 1 *versus* light bulb in room 2). However not all appliances are equipped with SMPS and noise signatures are sensitive to wiring architecture. Furthermore, the study has ignored the EM interference sources in the surrounding environment that could affect the performance of the system. We provide a summary of most significant transient-state NILM methods in Table 2 and highlights the advantages and disadvantages of different approaches.

### 3.3. Non-Traditional Appliance Features

**Figure 4.**
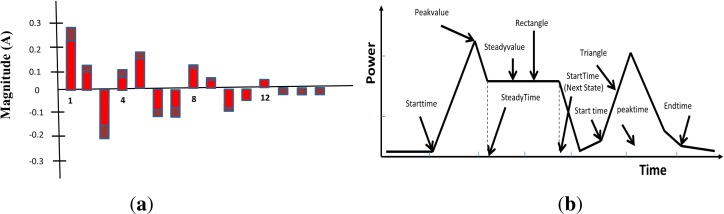
(**a**) Harmonic signature of monitor where black bars show fluctuations, (From [17]); (**b**) Schematic diagram of two unit graph, (From [8]).

Apart from traditional steady-state and transient analysis, recently we have seen an increased interest in feature extraction methods to acquire non-traditional appliance features. Recently in [8], the author has proposed that the power consumption of residential appliances can be described by the combination of two basic units, rectangles and triangles, neglecting the smaller fluctuations and errors. It has been argued that this new approach can reduce the problem of appliance feature overlap. The triangle unit can be expressed by *starttime*, *peaktime*, *peakvalue* and *endtime* whereas the rectangle can be described by *starttime*, *peaktime*, *peakvalue*, *steadytime*, *steadypower* as shown in Figure 4(b). Mean-shift clustering method is used to quantify the proposed units that define the working style of different categories of appliances. The load identification process combines the fundamental features with working styles to classify appliances with an accuracy of 80%. The major advantage of this method is that it does not require any training or supervision. Liang *et al.*[18] proposed to combine several features including *P*, *Q*, harmonics of the appliances as shown in Figure 4(a), eigenvalues of the current waveforms, admittance etc for load disaggregation. It has been argued that combination of features improves the performance of load identification algorithms. Suzuki *et al.*[35] have tried to examine the use of raw waveforms for appliance identification, however the experimental evaluations showed that it offers no advantage whereas in comparison the processed features are better suited for load identification. Other non-traditional features including time of the day, on and off duration distribution, frequency of appliance usage as well correlation between the usage of different appliances have also been examined by the researchers [49,50] to improve the performance of disaggregation algorithms.

## 4. Learning and Inference in NILM Systems

As mentioned earlier in Section 2, based on current literature, the supervised disaggregation methods for NILM systems can broadly be divided into optimization or pattern recognition based algorithms. The supervised learning mechanism requires labeled data sets to train the classifier so it would be able to recognize appliance operations from the aggregated load measurement. However, system training requires setting up initial instrumentation, which incurs extra cost and human effort. Therefore, lately researchers are actively looking to devise completely unsupervised or semi-supervised methods that can reduce the effort of acquiring the training data. In this section, we review the supervised and unsupervised learning methods for load disaggregation and further discuss their limitations and corresponding challenges.

### 4.1. Supervised Learning Approaches

**Optimization Methods:** Optimization based methods deal with the task of load disaggregation as an optimization problem. In the case of single load recognition, it compares the extracted feature vector of an unknown load to that of known loads present in the pool of the appliance database and tries to minimize the error between them to find the closest possible match. It can be mathematically expressed as:

(2) $$\mathrm{class}=\underset{i}{\text{argmin}}\left\|{\hat{y}}_{i}-y_{i}\right\|$$

where the *ŷ_i_* is the appliance feature available in the signature library and the *y_i_* is the new feature extracted due to occurrence of an unknown event. However, the optimization problem becomes more complex in case of composite load disaggregation as now instead of a one-to-one matching the algorithm has to take into account possible combination of appliances present in the known database, which could have generated the observed signal. Researchers [7,18,20,35] have tried different optimization approaches including integer programming and genetic algorithms in order to tackle the optimization problem. However it becomes a challenge to reduce the complexity of these methods especially if any unknown loads (*i.e.*, which are not included in the database) are present in the aggregated load data. Secondly, apart from being computationally expensive, appliances with similar or overlapping load signature are difficult to discern using this approach.

**Pattern Recognition Methods:** Pattern matching approaches are the ones most frequently used by the researchers for load disaggregation. The appliance database contains multiple appliance specific features that are used to define the structure and parameters of the recognition algorithm. A simple clustering based approach is proposed by Hart [6] in which appliances form their unique clusters in the *P*-*Q* plane. For load identification, the steady-state changes of the electrical signal are mapped to a feature space. In the next step, clustering analysis based on the distance metric is performed to identify if the new feature vector belongs to one of the known clusters. Due to the simplicity of the method, it has been widely applied in the NILM research however the inability of the algorithm to recognize appliances with overlapping *P*-*Q* features and sensitivity to power drifts are few of the major drawbacks. To address these problems, researchers [26,34,51,52] have extended this method to improve the load disaggregation performance. In [26], filtering and smoothing mechanisms have been used to deal with power variations and instead of power consumption change in real power values are used as a feature to detect appliances, however this approach only considers high power loads and furthermore it requires excessive training.

In [12] the author makes use of the Bayesian approach to detect most likely states of the appliances using *P* and state-change information. For each individual device, a naïve Bayes classifier has been trained and accordingly a set of trained classifiers have been used to recognize appliance-specific states from the aggregated load measurements. However, an assumption was made that the state of the appliances are independent of each other, which we believe is not true as it can easily be seen in the residential environment that the operation of consumer appliances are often correlated (e.g., the use of DVD player and a television). The Bayesian approach has been compared against a heuristic method that makes use of the histogram thinning technique to cluster *P* and *Q* events from the measurements. It was demonstrated that the Bayesian approach performs better than the heuristic method especially if the appliances have stable power behavior. However, only a handful of appliances have been used for experimental evaluations. Oppositely, the number of appliances to be monitored by a real-world NILM system could be very large. At the same time, the low-power consumer appliances often have subtle difference in their power signatures [53], therefore high load recognition accuracy could only be achieved if the target appliances have clear separation of features in the feature space.

Researchers have shown that the temporal information in combination with real power values can facilitate the load disaggregation algorithms [5,54]. Therefore Artificial Neural Networks (ANN) [37] and Hidden Markov Models (HMM) [55], have shown to perform well for the task of load disaggregation due to their ability to incorporate in their learning, temporal as well as appliance state transition information. The complexity of the HMM models, however, exponentially increases as the number of target appliances increases, which limits the applicability of this learning method. Besides, if any new appliance class has to be added, the complete model needs to be retrained each time. Alternatively, ANN [37] offers better extensibility and model performance can further be improved through a feedback input, however it requires exhaustive training for each appliance. On the other hand, Support Vector Machines (SVM) have shown good performance in classifying appliances especially using harmonic signatures and low frequency features as reported in [17,33,54]. A hybrid SVM/GMM model has recently been proposed by [53] in which GMM is used to describe the distribution of current waveforms, so as to find power similarity; while an SVM performs classification on the extracted power features in order to recognize operations of target loads. It can be seen from the literature that while few disaggregation algorithms are found out to be robust, even if they are exposed to unknown load signatures, oppositely others fail to cope with a situation even if the interclass variability of load signatures is high. Recently, Liang *et al.*[18,56] have proposed to combine different algorithms as well as appliance features using committee decision mechanisms (CDM) to improve the overall disaggregation accuracy. It is important to mention that the performance of the above mentioned classifiers are highly dependent on the feature sets, the type and number of target appliances being used in the experimentation. Therefore, a direct and fair performance comparison of different classifiers cannot be made unless a reference dataset is used in the evaluation, as discussed in Section 5.

### 4.2. Unsupervised Learning

Recently researchers have started to explore methods to achieve disaggregated energy sensing without *a-priori* information. It is highly desirable for the NILM systems to be installed in a target environment with a minimal setup cost as the training requirement for the supervised load identification algorithms is expensive and laborious. Hence, unsupervised learning approaches are needed for a wider applicability of NILM techniques.

**Figure 5.**
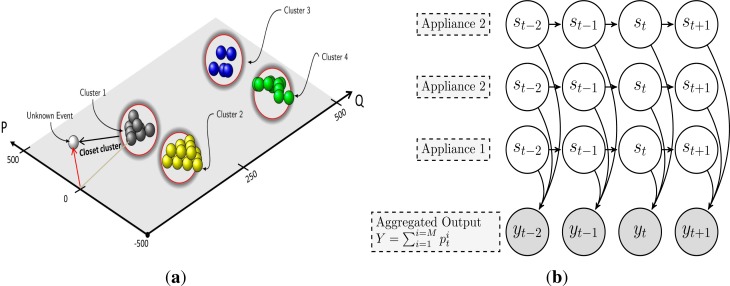
(**a**) MP algorithm tries to match the unknown event with the closest possible source; (**b**) To define a combined load model, appliance HMM’s are arranged in a specialized structure to form a Factorial HMM.

In [57], a blind source separation technique has been applied to discern appliances from the aggregated load data in an unsupervised fashion. The steady-state Δ*P* and Δ*Q* features have been used to cluster appliances. The Genetic K-means and agglomerative clustering approaches have been investigated to automatically determine the total number of appliance clusters from the load data. Each cluster is assumed to be a linear combination of multiple appliance sources, which are further broken down into individual sources. The matching pursuit (MP) is used for source reconstruction, whereas the algorithm iteratively tries to minimize the distance between the unknown event and the possible clusters as shown in Figure 5(a). However, there are several drawbacks and challenges as highlighted by the author himself; firstly most of the false negative events are generated by the smaller appliances especially the kitchen lights, due to the similarities in the consumption level. Secondly, in the case of multi-state appliances, source reconstruction becomes even more challenging as they form several clusters due to multiple states, which results in mixing of the events. On the other hand, large residential appliances can be easily detected as they form separate clusters. The study has shown that the genetic k-means approach has performed better than the agglomerative based method.

Recently, [58] has proposed to use the motif mining approach for unsupervised energy disaggregation. In order to recognize individual appliances, power change events such as (+500 W, −500 W) have been considered in contrast to power consumption. The motif mining approach has been used to identify recurring events referred to as episodes that is basically the On/Off operation of the devices. Each episode must fulfill certain conditions so that it can be considered as interpretable, and finally minimal episode completion criteria is imposed in order to identify only those episodes that are completed by a single device. While the approach is feasible for appliances that have a repeatable and distinctive events but it is not well understood how this approach will deal with appliances having variable consumption pattern as well devices with similar episodes. Kim *et al.*[49] developed probabilistic models of appliance behavior using variants of Factorial HMM (FHMM). The non-power features such as duration and time of appliance usage along with their real power consumption are used to model device specific HMMs. The aggregated load data (*Y*) at any point in time *t* depends on the power drawn by the appliances operating in their particular states as shown in Figure 5(b). FHMM on the other hand is well suited to model the interaction of several processes such as appliances contributing independently to the aggregated power measurements. Therefore, given *Y* the task is to find the best possible hidden state sequence (*q*) that might have resulted the observation. The parameters of the model are learned using Expectation Maximization (EM) algorithm, and Gibbs sampling is used to find the best possible q*:

(3) $$q*=\underset{q}{\text{argmax}}P(Y,q|\lambda )$$

In comparison with other FHMM models, Conditional Factorial Hidden Semi-Markov Model (CFHSMM) showed the best unsupervised disaggregation performance achieving an accuracy of 83%. The analysis of power measurements gathered from seven different homes showed that the On-state occupancy distribution of the devices can be best modeled using gamma distribution. The inclusion of these non-power features as well as additional information such as correlation between usage of appliances has shown to improve the overall disaggregation performance. However, the model performance decreases as the number of target appliances increases. At the same time, the reported work only considers the binary operation of the appliances, there is no discussion on how to estimate the total number of appliances from the observed data and how the inclusion of unknown appliances may affect the model performance.

Another drawback of the FHMM based approach is that existing inference techniques for hidden state estimation are highly susceptible to local optima. To address this issue, [59] has proposed a new inference algorithm, Additive Factorial Approximate MAP (AFMAP), with a convex formulation. It has been applied for the task of unsupervised energy disaggregation and used to perform inference over the additive FHMM’s in order to separate appliances from the aggregated load data. In comparison with previous inference methods, the author showed that the proposed formulation works better but only the precision and recall measures have been reported as the true contribution of each appliance from the circuit was unknown. The frequently occurring appliance patterns similar to [58] are used as load signature to model the HMMs. AFMAP discern appliances with an average precision of 87%, however the precision for electronics and kitchen outlets is less than 50%. Besides, the evaluations include limited devices with only short time scale of operation; however there is a possibility to extend the model to incorporate more appliances.

**Figure 6.**
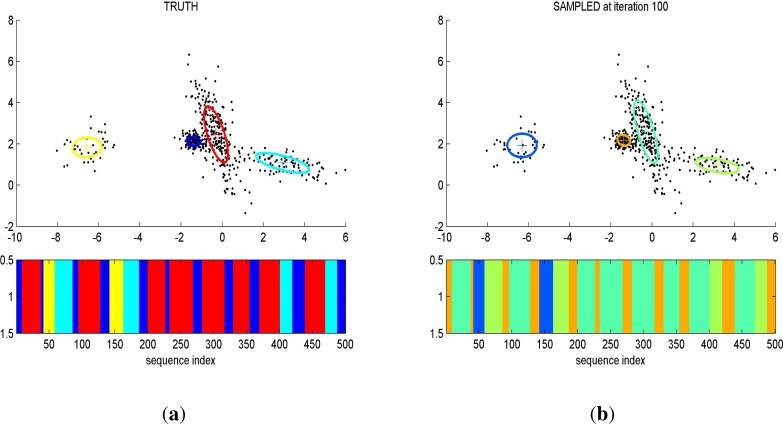
(**a**) Data generated from the true model; (**b**) Model Learned from the data using HDP-HSMM: Reproduced results from [60].

In [60] the author has applied Hierarchical Dirichlet Process Hidden Semi Markov Model (HDP-HSMM) factorial structure to the problem of unsupervised power disaggregation. The HDP-HSMM has the ability to incorporate duration distributions that allow it to learn from complex sequential data as shown in Figure 6. This technique addresses some of the drawbacks of previous approaches such as in contrast to [49,59] it is not limited to the binary states of the appliances and instead of using training data to learn the parameters; it learns the device models during inference process. This approach provides an advantage over EM-based learning methods as it is not only faster in terms of speed but also provides flexibility in learning the device models, whereas the Expectation Maximization (EM) based device models are dependent on the training data, which may not be a consistent across all homes. To build appliance models, the change points in the data must be identified and grouped to represent a complete operation, which again leads to some of the inherent problems faced by NILM methods, including similarity in the change points of multiple appliances as well as an inability of the algorithm to identify small loads in the presence of large loads.

Finally, we summarize the comparison of most commonly used learning algorithms for load disaggregation in Table 3.

## 5. Performance Evaluation of Load Disaggregation Algorithms

Recognition accuracy is the most widely used performance evaluation metric for accessing the accuracy of learning algorithms. Most of the research work in NILM report the performance of their system using accuracy metrics. However, due to inconsistency in the definition of accuracy it is not possible to draw meaningful comparisons between reported research work [5]. The overall accuracy measure is not a suitable metric particularly for the multi-class classification problem because it is impaired with data unbalance issue. Therefore, researchers often report a Confusion Matrix (CM) to provide an insight into model performance. Liang *et al.*[18] have addressed this issue and suggested three accuracy measures to be used for performance evaluation: Detection accuracy, disaggregation accuracy, and overall accuracy. Researchers working in the field of pattern recognition often use Receiver operating Curves (ROC) to compare the performance of different models. It has been suggested by [5] that ROC curves could also be used as a reference evaluation method to benchmark NILM algorithms. Another recent work [63] has proposed and discussed metrics for the evaluation of load disaggregation algorithms specifically for event detection approaches.

Apart from a common evaluation metric there is also a lack of reference dataset on which the performance of algorithm can be compared. It is quite obvious that the output of the load disaggregation algorithm is dependent on the source data, which often varies either due to difference in the number and type of appliances used in the experiment or due to the hardware used to extract the load signatures. In order to draw meaningful performance comparison of various NILM techniques, the availability of common datasets is critical. Motivated by this, recently the Reference Energy Disaggregation Data Set (REDD) [28] and the Building-Level fUlly labeled Electricity Disaggregation dataset (BLUED) [64] have been made publicly available in order to facilitate the researchers in the development and evaluation of new load disaggregation algorithms. The datasets contain high-frequency and low-frequency household power measurements primarily for the evaluation of steady-state as well as transient state NILM methods. In a similar context a *UMass* Smart* Home Dataset [65] which is part of a Smart* project has also been released and, in contrast with previous examples, this particular dataset not only provides power measurements but also includes heterogeneous sensory information (*i.e.*, motion, thermostat, door and wall switch events) acquired from the target homes. The data from the environmental sensors can be very useful for analyzing the correlation between non-power events and the power event, which could foster the research in the design of multi-modal sensing framework as discussed in Section 6. To address some of the prevailing challenges in the field of NILM, it is critical that researchers adopt common evaluation metrics and datasets, so that various load disaggregation approaches can be fairly compared.

## 6. Challenges and Future Research Directions

In the previous sections we have discussed existing NILM methods, highlighting the gaps and limitations of current approaches. We will summarize those challenges in the discussion below while pointing out important future research directions.

### 6.1. Beyond Traditional NILM Approaches

Recently researchers have started to investigate the usefulness of using external sources of information with power-centric appliance features. It has been demonstrated in [66] that operation of appliances not only impact the power stream but in parallel they also generate environmental information which can be captured and further exploited to improve the accuracy of load disaggregation algorithms, particularly in the case of low-power appliances. For example, it has been shown that one can achieve better disambiguation between a kitchen light and an overhead light with similar power traces, just by using additional information achieved from a light sensor placed in the bathroom. Additionally, leveraging data from environmental sensors can also assist in training of the supervised disaggregation algorithms. The measurements of light intensity, temperature and motion can be correlated with appliance operating states to facilitate automated training procedures, as well as identifying appliances with similar consumption patterns. The labeling of training data required by the load disaggregation algorithms is one of the major challenges faced by single point sensing solutions as it requires human effort and intervention. At present, in order to acquire appliance signatures users have to manually switch on or off each appliance one by one, so that manual labellings of the devices can be done in the software, this is time consuming and error-prone.

ANNOT [21] is a prototype system that has been developed by the researchers to automate the data annotation process for the NILM systems. The proposed solution detects and labels appliances using acoustic signatures and indirect power sensing. The system correlates heterogeneous-sensory information with the power measurements in time to label the appliances without the need of human supervision. Apart from automatic data annotation, this method offers a possibility to validate system results by matching the output of learning algorithm with that of annotated training data. In order to test the feasibility of the approach, a single point sensing solution, the RECAP system [37] has been employed as already discussed in Section 3. Originally, RECAP requires a human supervision for developing an appliance feature database. Subsequently, it requires manual effort to assign labels to the features in the database, but the integration of ANNOT with the RECAP system removes any need for the user during the labeling process. The combination has achieved high accuracy in detecting appliances, however the drawback is the additional cost associated with developing an automatic annotation system.

The multi-modal sensing framework as already reported in [21,66,67] will benefit not only NILM approaches, but it will enable the energy management solutions to look beyond quantification of energy consumption only. In [68] it has been demonstrated that in addition to energy monitoring, possible scenarios of energy wastage can also be identified by combining information from Electromagnetic Field Detector (EMF) based appliance state detector developed by CMU researchers [22] and the motion sensors. Zoha *et al.* have proposed a multi-modal sensing architecture that fuses information from sound and an energy meter to minimize the ambiguous overlapping of power features in the *P*-*Q* plane. Similarly, in order to obtain an accurate estimate of the device’s energy consumption pattern, the author in [69] has proposed a design of an energy monitoring system that correlates energy meter readings and sound samples acquired from the acoustic sensors to identify the operational state of the appliances. However, these approaches demand the additional installation of sound sensors to detect machine sounds from the target environment. To avoid the installation of sound sensors, a real-time prototype system has been developed in [70] that makes use of a microphone sensor available on a mobile phone in order to fingerprint the profile of each individual machine. The results from this initial study not only look promising but the concept of using mobile phones for disaggregated energy sensing is also very interesting. However, the evaluations do not consider simultaneous operation of appliances and the locations and layout of the machines have assumed to be already known.

At the same time, contextual information has also been utilized with power features for load disaggregation. Initially, context is defined as just the location of the user however this definition has evolved in the past years incorporating many aspects of user environment. In the realm of ALM, we found the most relevant definition to be: *“Context is the information that can be used to characterize the situation of entities i.e., whether a person, place or object) that are considered relevant to the interaction between a user and an application, including the user and the application themselves”*[71]. Location however is a key context feature because it represents the user’s position, which helps a system to infer possible services and functionalities. In [67] authors propose a method that takes user location information acquired through wireless sensor network, and use it to enhance the performance of load disaggregation algorithm. The load state changes are correlated with the user location whereas it has been shown that the proposed algorithm was able to correctly discern appliances with overlapping load signatures. However, for location estimation it has been assumed that the user carries a sensor whose signal strength is tracked to locate user’s position. Similarly, Harris and Cahill [72] detects user location and identify whether the user is in a vicinity of a device with the help of Bluetooth and a microphone. The location information is used to develop Context-Aware Power Management (CAPM) system to control the states of appliances, automatically configuring them to different modes for optimal power utilization.

The two attributes *“Time of the day”* as well as the “*appliance usage duration*” have also been used in combination with load signatures to enhance the performance of load disaggregation algorithms. It has been shown in [49,73] that these non-power features play a decisive role in discriminating between appliances with similar load characteristics particularly in the case of learning algorithms that perform appliance classification in an unsupervised manner. Furthermore, these two features can also facilitate energy companies to formulate time-of-use pricing policies. Moreover, the frequency of appliance use is also considered as a feature in [49,74] where researchers try to find a relationship between activities and device usage by measuring which devices are most frequently used in several activities. It has been found out that the activity of a user has a direct relationship with a device or a group of devices; hence keeping in mind energy conservation goals the purposeless use of such devices can be detected so that users can be informed about it. It is because the major use case for NILM system is not just to facilitate consumers by providing a detail break down of their energy expenditure, but identification of load patterns, precise measurement of energy demands, personalized billing, fault monitoring, and tailored energy feedback are few of the application areas that can directly benefit from it. [70]

In [75] the author suggests that devices should be grouped based not on the level of their automation but on the level of user presence and control required for the operation of the device. A similar project by Microsoft [76] employs energy saving strategies by relating user presence with the appliance usage. The presence of the user is detected via motion sensors installed in the living areas and based on the occupancy status electricity saving is achieved by controlling the devices (consumer electronics, lightning, heating) accordingly. In [77] researchers have tried to utilize context information (*i.e.*, user presence) to optimally control HVAC systems in a building. It is important point to highlight that although the use of external sensing modalities facilitates the current NILM approaches and in contrast with previous examples it introduces additional challenges as faced by ILM approaches such as cost and installation complexity. We believe the way forward in the future is to remain minimally intrusive by utilizing the opportunistically available resources such as mobile phones, security sensors installed within homes and buildings, *etc*.

### 6.2. Challenges

Even after two decades, still there are numerous challenges that need be addressed in order to make NILM a practically viable solution. It is still a challenge to develop a solution that could perform well in discerning all types of appliances regardless of their category, make, size and the manufacturer. It is hard to form generic appliance models due to high interclass variability of features within each load category whereas the power draw pattern by most of the multi-state appliances is dependent on user-specific settings. Moreover, there are no widely accepted load signatures that can model well the operation of all three categories of appliances. As mentioned in Section 5 due to the lack of reference datasets it was not possible to perform fair comparisons and testing of different appliance feature sets. Additionally, low power consumer appliances exhibit similar power consumption characteristics making the recognition task even more challenging. As most of the work in NILM is based on supervised learning, which requires each appliance to be profiled during the training phase, a subtle change by an energy supplying company (*i.e.*, power factor correction) at the main circuit line can cause a mismatch of appliance profile [37]. Another closely related challenge for NILM is the update of the appliance signature database. We can easily conclude from our literature review that most of the NILM solutions for load disaggregation require off-line training of the algorithms. It involves building a database for appliance signatures that in turn is limited to the appliances being used as exemplars, which is further used to train the algorithms for classification. It is impractical to include all the appliances of different characteristics (make, model, size, *etc*.) in the database and hence algorithms would not be able to recognize any such device that is not in the appliance signature database. The question of how to identify new devices that are not included in signature database still needs an answer. One possible solution is to make use of interactive technology in which a system can interact with the user using different interaction channels such as WEB and through mobile phone based short message service. In the presence of an unknown load the system makes an intelligent guess or the closest match will be presented to the user for verification. The user input can further be used to label the device pattern and consequently the appliance signature library will be updated.

Future work should focus on unsupervised learning algorithms for NILM that do not require human labeling of data. In order to facilitate the grouping and detection of appliances in an unsupervised fashion, the contextual cues from the environment and device usage patterns can further be exploited. The algorithms including KNN [78], ISODATA [79], Self-Organizing Trees [80] do not require a pre-learning phase and they have been successfully employed in other research domains for the task of unsupervised pattern classification. The effectiveness of these learning methods for unsupervised load disaggregation can further be explored. For commercialization of NILM systems cost is a key factor, therefore researchers are trying to improve the performance of steady-state methods by combining them with context-aware features such as time of use and frequency of use information. However, there is a trade-off between cost and information because in order to detect parallel device activity or simultaneous load activations high frequency measurements are inevitable. Lastly, there are privacy concerns associated with ALM approaches as addressed in [81].

**Figure 7.**
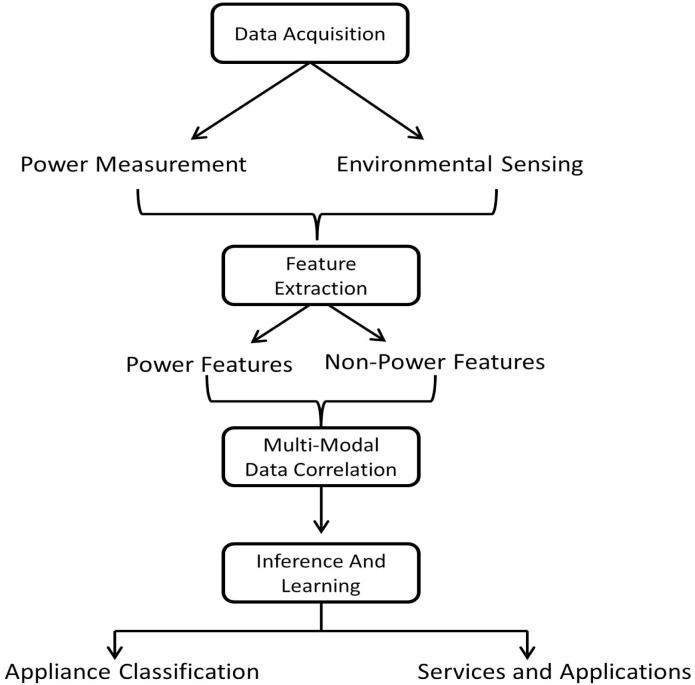
Multi-Modal Sensing Framework for NILM based load disaggregation.

It has already been discussed above that NILM can take advantage of distributed sensing architecture not only for improving the load disaggregation performance, but to address some major challenges such as unsupervised learning and inference, formation of generic appliance models as well to achieve a better understanding of user consumption behavior. We believe the research in NILM methods is moving towards a multi-modal sensing framework as shown in Figure 7, that could possibly answer the prevailing challenges and offer ease of integration to other research areas in future.

## 7. Conclusion

Due to high cost and intrusive nature of ILM based methods, the research in the field of ALM is more focused toward non-intrusive approaches. NILM shows promising results in measuring appliance-specific energy consumption, while keeping installation cost and complexity low. We have presented a review of NILM methods that make use of steady-state and transient load signatures in combination with state-of-the art load disaggregation algorithms. Our review draws several conclusions:

1. No set of appliance features as well as load disaggregation algorithms are found to be suitable for discerning all types of appliances.
2. To date, too little attention has been paid in devising a method for automatic data annotation systems. On the other hand, the pre-deployment setup to acquire the training dataset for the supervised load disaggregation algorithms is not only expensive but also is not practical. Therefore, we believe that research in the future should focus on unsupervised learning methods because that would not only make NILM systems inexpensive and easy to install but would allow wider adoption.
3. The heterogeneous sensory information in a multi-modal sensing framework can further be exploited to address some of the prevailing challenges faced by the current NILM techniques, however at the cost of increased complexity. The ambiguous overlapping of appliance features, especially for the case of low-power appliances, as well as the issue of high interclass variability of appliance features can be addressed by combining power and non-power features. In addition, the multi-modal framework could open opportunities for the integration of other research domains, for example NILM systems can facilitate ubiquitous computing applications such as user activity recognition.
4. Finally, the adoption of standard evaluation procedures for measuring the performance of NILM methods is critical for the advancement of research in disaggregated energy sensing.

## Figures and Tables

**Table 1.** Summary of steady-state methods.

**Steady-State Methods**	**Features**	**Advantages**	**Shortcomings**
Power Change [6,12,13,26,30]	Steady State Variation of Real and Reactive Power, Δ*P*, Δ*Q*	High-Power Residential Loads can easily be identified, Low-sampling rate requirement,	Low power appliances overlap in P-Q plane, Poor performance in recognizing Type-II, III and Type-IV loads.
Time and Frequency Domain Characteristics of VI Waveforms [18,32–38]	Higher order Steady-State Harmonics, Irms, Iavg,Ipeak, Vrms, Power factor	Device classes can easily be categorized into resistive, inductive and electronic loads	High sampling rate requirement, Low accuracy for Type-III loads, overlapping features for consumer electronics of Type-I and II category, unable to distinguish between overlapping activation events
V-I Trajectory [39,40]	Shape features of V-I trajectory : asymmetry, looping direction, area, curvature of mean line, self-intersection, slope of middle, segment, area of segments and peak of middle segment	Detail taxonomy of electrical appliances can be formed due to distinctive V-I curves	Sensitive to multi-load operation scenario, computationally intensive, smaller loads have no distinct trajectory patterns
Steady-State Voltage Noise [11,41]	EMI signatures	Motor-based appliances are easily distinguishable as they generate synchronous voltage noise, Detection of simultaneous activation events, Consumer appliances equipped with SMPS can be recognized with high accuracy	Sensitive to wiring architecture, EMI signatures overlap, Not all appliances are equipped with SMPS

**Table 2.** Summary of transient-state methods.

**Transient Methods**	**Features**	**Advantages**	**Shortcomings**
Transient Power [5,36,43,47,48]	Repeatable transient power profile, spectral envelopes	Appliances with same power draw characteristics can be easily differentiated, Recognition of Type I,II,III loads	Continuous monitoring, high sampling rate requirement, not suitable for Type IV loads
Start-Up Current Transients [13,44,47]	Current spikes, size, duration, shape of switching transients, transient response time	Works well for Type I and II loads, distinct transient behavior in multiple load operation scenario	Poor detection of simultaneous activation deactivation of sequences, unable to characterize Type III and IV loads, sensitive to wiring architecture, appliance specific
High Frequency Sampling of Voltage Noise [11,31]	Noise FFT	Multi-state devices, consumer Electronics with SMPS	Appliance specific, computationally expensive, Data annotation is very hard

**Table 3.** Comparison of load disaggregation algorithms.

**Learning Algorithm**	**Features St^a^/Tr^b^**	**Accuracy %**	**Training S^c^/U^d^**	**Online/Offline**	**Scalability**	**Appliance Types**
SVM [11,17,33,54]	B ^e^	75–98	S	Online	Yes	I, II, III & IV
Bayes [12,50,54]	St	80–99	S	B	No	I & II
HMM [49,59,60]	St	75–95	B	Offline	No	I & II
Neural Networks [17,37,61]	B	80–97	S	Online	Yes	I & II & III
KNN [6,9,62]	B	70–90	S	B	Yes	I & II
Optimization [7,18,20,35]	St	60–97	S	Offline	No	I & II

a Steady-State

b Transient

c Supervised

d Unsupervised

e Both.

## References

[1] Uteley J., Shorrock L. (2008). Domestic Energy Fact File 2008.

[2] Darby S. (2006). The Effectiveness of Feedback on Energy Consumption: A Review for Defra of the Literature on Metering, Billing and Direct Displays.

[3] Ehrhardt-Martinez K., Donnelly K.A., Laitner J.A. (2010). Advanced Metering Initiatives and Residential Feedback Programs: A Meta-Review for Household Electricity-Saving Opportunities.

[4] (2010). Energy Consumption in United Kingdom.

[5] Zeifman M., Roth K. (2011). Nonintrusive appliance load monitoring: Review and outlook. IEEE Trans. Consum. Electron.

[6] Hart G.W. (1992). Nonintrusive appliance load monitoring. IEEE Proc.

[7] Baranski M., Voss J. Non-Intrusive Appliance Load Monitoring Based on an Optical Sensor.

[8] Wang Z., Zheng G. (2012). Residential appliances identification and monitoring by a nonintrusive method. IEEE Trans. Smart Grid.

[9] Berges M., Goldman E., Matthews H.S., Soibelman L., Anderson K. (2011). User-centered non-intrusive electricity load monitoring for residential buildings. J. Comput. Civil Eng.

[10] Carrie Armel K., Gupta A., Shrimali G., Albert A. (2013). Is disaggregation the holy grail of energy efficiency? The case of electricity. Energ. Policy.

[11] Patel S.N., Robertson T., Kientz J.A., Reynolds M.S., Abowd G.D. At the Flick of a Switch: Detecting and Classifying Unique Electrical Events on the Residential Power Line.

[12] Marchiori A., Hakkarinen D., Han Q., Earle L. (2011). Circuit-level load monitoring for household energy management. IEEE Pervas. Comput.

[13] Norford L.K., Leeb S.B. (1996). Non-intrusive electrical load monitoring in commercial buildings based on steady-state and transient load-detection algorithms. Energ. Build.

[14] Leeb S.B., Shaw S.R., Kirtley J.L. (1995). Transient event detection in spectral envelope estimates for nonintrusive load monitoring. IEEE Trans. Power Del.

[15] Jin Y., Tebekaemi E., Berges M., Soibelman L. Robust Adaptive Event Detection in Non-Intrusive Load Monitoring for Energy Aware Smart Facilities.

[16] Cox R., Leeb S., Shaw S., Norford L. Transient Event Detection for Nonintrusive Load Monitoring and Demand Side Management Using Voltage Distortion.

[17] Srinivasan D., Ng W., Liew A. (2006). Neural-network-based signature recognition for harmonic source identification. IEEE Trans. Power Del.

[18] Liang J., Ng S.K.K., Kendall G., Cheng J.W.M. (2010). Load signature study Part I: Basic concept, structure, and methodology. IEEE Trans. Power Del.

[19] Du Y., Du L., Lu B., Harley R., Habetler T. A Review of Identification and Monitoring Methods for Electric Loads in Commercial and Residential Buildings.

[20] Baranski M., Voss J. Genetic Algorithm for Pattern Detection in NIALM Systems.

[21] Schoofs A., Guerrieri A., Delaney D., O’Hare G., Ruzzelli A. ANNOT: Automated Electricity Data Annotation Using Wireless Sensor Networks.

[22] Rowe A., Berges M., Rajkumar R. Contactless Sensing of Appliance State Transitions through Variations in Electromagnetic Fields.

[23] Giri S., Berges M. A Study on the Feasibility of Automated Data Labeling and Training Using an EMF Sensor in NILM Platforms.

[24] Drenker S., Kader A. (1999). Nonintrusive monitoring of electric loads. IEEE Comput. Appl. Power.

[25] Kolter J.Z., Batra S., Ng A.Y. (2010). Energy disaggregation via discriminative sparse coding. Adv. Neural. Inform. Process. Syst.

[26] Farinaccio L., Zmeureanu R. (1999). Using a pattern recognition approach to disaggregate the total electricity consumption in a house into the major end-uses. Energ. Build.

[27] Park S., Kim H., Moon H., Heo J., Yoon S. (2010). Concurrent simulation platform for energy-aware smart metering systems. IEEE Trans. Consum. Electron.

[28] Kolter J.Z., Johnson M.J. REDD : A Public Data Set for Energy Disaggregation Research.

[29] Li Y., Trayer M., Ramakrishna S., Lai P.H. Training Schemes in Non-Intrusive Load Monitoring.

[30] Marceau M.L., Zmeureanu R. (2000). Nonintrusive load disaggregation computer program to estimate the energy consumption of major end uses in residential buildings. Energ. Convers. Manag.

[31] Hazas M., Friday A., Scott J. (2011). Look back before leaping forward: Four decades of domestic energy inquiry. IEEE Pervas. Comput.

[32] Najmeddine H., El Khamlichi Drissi K., Pasquier C., Faure C., Kerroum K., Diop A., Jouannet T., Michou M. State of Art on Load Monitoring Methods.

[33] Kato T., Cho H.S., Lee D. Appliance Recognition from Electric Current Signals for Information-Energy Integrated Network in Home Environments.

[34] Cole A., Albicki A. Nonintrusive Identification of Electrical Loads in a Three-Phase Environment Based on Harmonic Content.

[35] Suzuki K., Inagaki S., Suzuki T., Nakamura H., Ito K. Nonintrusive Appliance Load Monitoring Based on Integer Programming.

[36] Laughman C., Lee K., Cox R., Shaw S., Leeb S., Norford L., Armstrong P. (2003). Power signature analysis. IEEE Power Energ. Mag.

[37] Ruzzelli A.G., Nicolas C., Schoofs A., O’Hare G.M.P. Real-Time Recognition and Profiling of Appliances through a Single Electricity Sensor.

[38] Li J., West S., Platt G. Power Decomposition Based on SVM Regression.

[39] Lee W.K., Fung G.S.K., Lam H.Y., Chan F.H.Y., Lucente M. Exploration on Load Signatures.

[40] Lam H.Y., Fung G.S.K., Lee W.K. (2007). A Novel method to construct taxonomy electrical appliances based on load signaturesof. IEEE Trans. Consum. Electron.

[41] Gupta S., Reynolds M.S., Patel S.N. ElectriSense: Single-Point Sensing Using EMI for Electrical Event Detection and Classification in the Home.

[42] Figueiredo M., de Almeida A., Ribeiro B., Dobnikar A., Lotric U., Ter B. (2011). An experimental study on electrical signature identification of Non-Intrusive Load Monitoring (NILM) systems. Adaptive and Natural Computing Algorithms.

[43] Chang H.H., Yang H.T., Lin C.L., Shen W., Yong J., Yang Y., Barths J.P., Luo J. (2008). Load identification in neural networks for a non-intrusive monitoring of industrial electrical loads. Computer Supported Cooperative Work in Design IV.

[44] Cole A.I., Albicki A. Data Extraction for Effective Non-Intrusive Identification of Residential Power Loads.

[45] Lee K.D., Leeb S.B., Norford L.K., Armstrong P.R., Holloway J., Shaw S.R. (2005). Estimation of variable-speed-drive power consumption from harmonic content. IEEE Trans. Energy Convers.

[46] Wichakool W., Avestruz A.T., Cox R.W., Leeb S.B. (2009). Modeling and estimating current harmonics of variable electronic loads. IEEE Trans. Power Electron.

[47] Chang H.H. (2012). Non-intrusive demand monitoring and load identification for energy management systems based on transient feature analyses. Energies.

[48] Shaw S.R., Leeb S.B., Norford L.K., Cox R.W. (2008). Nonintrusive load monitoring and diagnostics in power systems. IEEE Trans. Instrum. Meas.

[49] Kim H., Marwah M., Arlitt M., Lyon G., Han J. Unsupervised Disaggregation of Low Frequency Power Measurements.

[50] Zeifman M. (2012). Disaggregation of home energy display data using probabilistic approach. IEEE Trans. Consum. Electron.

[51] Berges M., Goldman E., Matthews H.S., Soibelman L. Learning Systems for Electric Consumption of Buildings.

[52] Berges M.E., Goldman E., Matthews H.S., Soibelman L. (2010). Enhancing electricity audits in residential buildings with nonintrusive load monitoring. J. Ind. Ecol.

[53] Lai Y.X., Lai C.F., Huang Y.M., Chao H.C. (2012). Multi-appliance recognition system with hybrid SVM/GMM classifier in ubiquitous smart home. Inform. Sci..

[54] Lin G.y., Lee S.C., Hsu Y.J., Jih W.R. Applying Power Meters for Appliance Recognition on the Electric Panel.

[55] Zia T., Bruckner D., Zaidi A. A Hidden Markov Model Based Procedure for Identifying Household Electric Loads.

[56] Liang J., Ng S., Kendall G., Cheng J. (2010). Load signature study-part II: Disaggregation framework, simulation and applications. IEEE Trans. Power Del.

[57] Goncalves H., Ocneanu A., Bergés M., Fan R.H. Unsupervised Disaggregation of Appliances Using Aggregated Consumption Data.

[58] Shao H., Marwah M., Ramakrishnan N. A Temporal Motif Mining Approach to Unsupervised Energy Disaggregation.

[59] Kolter J.Z., Jaakkola T. (2012). Approximate inference in additive factorial HMMs with application to energy disaggregation. J. Mach Leran. Res.

[60] Johnson M.J., Willsky A.S. Bayesian Nonparametric Hidden Semi-Markov Models. http://arxiv.org/abs/1203.1365.

[61] Chan W., So A., Lai L. Wavelet Feature Vectors for Neural Network Based Harmonics Load Recognition.

[62] Saitoh T., Aota Y., Osaki T., Konishi R., Sugahara K. (2010). Current sensor based home appliance and dtate of appliance recognition. SICE J. Contr. Meas. Syst. Integrat.

[63] Anderson K., Berges M., Ocneanu A., Benitez D., Moura J.M.F. Event Detection for Non Intrusive Load Monitoring.

[64] Anderson K., Ocneanu A., Benitez D., Carlson D., Rowe A., Berges M. BLUED: A Fully Labeled Public Dataset for Event-Based Non-Intrusive Load Monitoring Research.

[65] Barker S., Mishra A., Irwin D., Cecchet E., Shenoy P., Albrecht J. Smart*: An Open Data Set and Tools for Enabling Research in Sustainable Homes.

[66] Berges M., Soibelman L., Matthews H.S. Leveraging Data from Environmental Sensors to Enhance Electrical Load Disaggregation Algorithms.

[67] Yoo J., Park B., Hur K. Context Awareness-Based Disaggregation of Residential Load Consumption.

[68] Berges M., Rowe A. Poster Abstract: Appliance Classification and Energy Management Using Multi-Modal Sensing.

[69] Guvensan M.A., Taysi Z.C., Melodia T. (2012). Energy monitoring in residential spaces with audio sensor nodes: TinyEARS. Ad Hoc Networks.

[70] Uddin M., Nadeem T. EnergySniffer: Home Energy Monitoring System Using Smart Phones.

[71] Dey A., Abowd G., Salber D. (2001). A conceptual framework and a toolkit for supporting the rapid prototyping of context. Hum-Comput. Interact.

[72] Harris C., Cahill V. Exploiting User Behaviour for Context-Aware Power Management.

[73] Powers J., Margossian B., Smith B. (1991). Using a rule-based algorithm to disaggregate end-use load profiles from premise-level data. IEEE Comput. Appl. Power.

[74] Lee S.C., Lin G.Y., Jih W.R., Hsu J.Y.J. Appliance Recognition and Unattended Appliance Detection for Energy Conservation.

[75] Wood G., Newborough M. (2007). Influencing user behaviour with energy information display systems for intelligent homes. Int. J. Energ. Res.

[76] Priyantha N.B., Kansal A., Goraczko M., Zhao F. Tiny Web Services: Design and Implementation of Interoperable and Evolvable Sensor Networks.

[77] Erickson V.L., Lin Y., Kamthe A., Brahme R., Surana A., Cerpa A.E., Sohn M.D., Narayanan S. Energy Efficient Building Environment Control Strategies Using Real-Time Occupancy Measurements.

[78] Weng F., Jiang Q., Chen L., Hong Z. Clustering Ensemble Based on the Fuzzy KNN Algorithm.

[79] Phillips S. Reducing the Computation Time of the Isodata and K-Means Unsupervised Classification Algorithms.

[80] Campos M.M., Carpenter G.A. (2001). S-TREE: self-Organizing trees for data clustering and online vector quantization. Neural Netw.

[81] Lisovich M., Mulligan D., Wicker S. (2010). Inferring personal information from demand-response systems. IEEE Secur. Privacy.

